# BetaScan2: Standardized Statistics to Detect Balancing Selection Utilizing Substitution Data

**DOI:** 10.1093/gbe/evaa013

**Published:** 2020-02-03

**Authors:** Katherine M Siewert, Benjamin F Voight

**Affiliations:** e1 Genomics and Computational Biology Graduate Group, Perelman School of Medicine, University of Pennsylvania; e2 Department of Systems Pharmacology and Translational Therapeutics, Perelman School of Medicine, University of Pennsylvania; e3 Department of Genetics, Perelman School of Medicine, University of Pennsylvania; e4 Institute for Translational Medicine and Therapeutics, Perelman School of Medicine, University of Pennsylvania

**Keywords:** balancing selection, selection scans, human evolution, selection statistics

## Abstract

Long-term balancing selection results in a build-up of alleles at similar frequencies and a deficit of substitutions when compared with an outgroup at a locus. The previously published $\beta^{\left(1\right)}$ statistics detect balancing selection using only polymorphism data. We now propose the $\beta^{\left(2\right)}$ statistic which detects balancing selection using both polymorphism and substitution data. In addition, we derive the variance of all *β* statistics, allowing for their standardization and thereby reducing the influence of parameters which can confound other selection tests. The standardized *β* statistics outperform existing summary statistics in simulations, indicating *β* is a well-powered and widely applicable approach for detecting balancing selection. We apply the $\beta^{\left(2\right)}$ statistic to 1000 Genomes data and report two missense mutations with high *β* scores in the *ACSBG2* gene. An implementation of all *β* statistics and their standardization are available in the BetaScan2 software package at https://github.com/ksiewert/BetaScan.

## Introduction

Balancing selection can maintain multiple alleles at a locus. Several scenarios can result in this type of selection, including heterozygote advantage or frequency-dependent selection. Recently, there have been significant methodological advances in the detection of balancing selection in population-level sequencing data (Siewert and Voight, 2017; DeGiorgio et al., 2014; Cheng and DeGiorgio, 2018; Bitarello et al., 2018). As a result of this recent interest, a number of specific balanced loci in a wide variety of species have been reported with strong observational or experimental evidence (Wheat et al., 2010; Network, 2015; Schweizer et al., 2018; Sano et al., 2018). These results suggest there may be more loci yet to be discovered.

Selection can maintain multiple balanced alleles for very long periods of time, referred to as long-term balancing selection. By doing so, selection increases the time to most recent common ancestor (TMRCA) (Charlesworth, 2006). The resulting signature can be detected to identify putatively balanced loci. Classically, this signature was defined as an excess of heterozygosity and a deficit of substitutions. This is due to variants building up around the balanced alleles through time but being prevented from fixing in the population (Hudson et al., 1987; Tajima, 1989). However, power analyses demonstrate that these classic methods have low power.

More recent methods detect a more precise signature in the site frequency spectrum, and as a result, are higher powered. Specifically, they detect a build-up of variants at highly similar frequencies (Siewert and Voight, 2017; Bitarello et al., 2018). This build-up is most likely a main contribution to the signal that is captured implicitly in methods that rely on simulations of balancing selection to generate an expected site frequency spectrum (DeGiorgio et al., 2014; Cheng and DeGiorgio, 2018). A build-up occurs because two allelic classes are maintained in a population, accumulating variation throughout time. These variants can fix within each haplotype class, and will therefore all be at the same frequency until their frequency is altered by recombination. This signature is not independent of excess heterozygosity, but is instead a more specific signature of the same underlying process.

Simulation-based methods have been shown to be the highest-powered methods, however they require additional types of data rendering them inapplicable in some situations (e.g., large grids of simulations of balancing selection, a sequenced genome from a closely related outgroup and/or genome-wide data in order to estimate the background site frequency spectrum; DeGiorgio et al., 2014; Cheng and DeGiorgio, 2018). In contrast, the $\beta^{\left(1\right)}$ statistics are nearly as high powered, yet do not require additional types of data, enabling wide-spread application.

The previously developed method *β* detects the signature of alleleic class build-up around each SNP it is applied to (i.e., each core SNP) (Siewert and Voight, 2017) by looking for an excess of genomically proximate SNPs that have a very similar allele frequency to the core SNP. Conceptually, *β* is a weighted sum of SNP counts around each core SNP, where SNPs are weighted more if they have very similar frequencies to the core SNP. By calculating a *β* score in a window around each SNP, one can find loci in which the pattern of allele frequencies is consistent with the action of balancing selection. Mathematically, *β* is the difference between two estimators of the mutation rate $\hat{\theta }$, one sensitive to excess frequency similarity (${\hat{\theta }}_{\beta }$) and one that is not (Watterson’s estimator ${\hat{\theta }}_{W}$) (Watterson, 1975). We previously reported two versions of *β*: $\beta^{\left(1\right)}$ incorporates outgroup sequence data to call ancestral allele states, whereas $\beta^{(1)*}$ only requires a folded site frequency spectrum.

We now report two improvements to *β*. The first is a new estimator based on the number of fixed differences with an outgroup species (i.e., substitutions), ${\hat{\theta }}_{D}$. Incorporating this estimator into our *β* framework increases power, owing to the expected reduction of substitutions under balancing selection (Hudson et al., 1987; Charlesworth, 2006). Second, we derive the variance of our statistics, enabling normalization of *β*. This allows *β* scores to be properly compared across a range of parameters which can affect its distribution, a feature lacking from other summary statistics, with the exception of Tajima’s *D* (Tajima, 1989).

## Results and Discussion

**Fig. 1.**
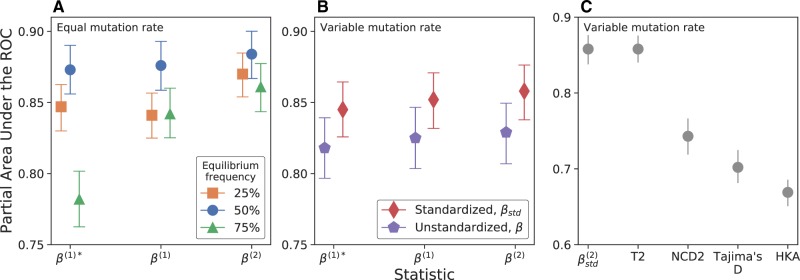
—Partial area under the receiver operator curve (ROC) from a false positive rate of 0 to 0.05 in simulations for each statistic under (*A*) different equilibrium frequencies and (*B*) with mutation rate variation, where one half of neutral and balanced simulation replicates had a mutation rate of $2.5\times 10^{-8}$ (our default rate), and the remaining half had a rate of $1.25\times 10^{-8}$. (*C*) Power of $\beta^{\left(2\right)}$ compared with other methods for detecting balancing selection. An equilibrium frequency of 50% was used for (*B*) and (*C*). The values of each statistic were compared between simulations containing only neutral variants (True Negatives) or with a balanced variant (True Positives). Confidence intervals show the 2.5th to 97.5th percentile for 1,000 sets of bootstrapped simulation replicates.

We measure the power of our statistics using simulations conducted using SLiM 2 (supplementary information, Supplementary Material online) (Haller and Messer, 2017). We find that $\beta^{\left(2\right)}$ has higher power than either $\beta^{\left(1\right)}$ statistic, with the greatest gain in power at equilibrium frequencies 25% and 75%, demonstrating that substitution counts provide additional signal over polymorphism data (fig. 1*A*, supplementary figs. 5 and 6,Supplementary Material online). When there is mutation rate variation across simulations, we find that standardization improves power (fig. 1*B*).

Next, we compare power with alternative methods: NCD2 (Bitarello et al., 2018), NCD_mid_ (Cheng and DeGiorgio, 2018), *T*1 and *T*2 (DeGiorgio et al., 2014), Tajima’s *D* (Tajima, 1989), and the HKA test (Hudson et al., 1987). *T*2 and *β* (fig. 1*C*) perform similarly. However, *β* does not require large grids of simulations as input and can be applied when an outgroup is not available. These two statistics significantly outperform NCD2, Tajima’s *D*, and the HKA test (fig. 1*C*, supplementary figs. 5 and 6, Supplementary Material online). Our results were not biased by window size (supplementary fig. 7, Supplementary Material online) or power comparison method (supplementary information, Supplementary Material online).

The power to detect balancing selection depends on underlying population parameters, including mutation rate, recombination rate and effective population size. We found that consistent with prior reports, the power of all methods increased with lower recombination rate (supplementary fig. 8, Supplementary Material online) and higher mutation rate (supplementary fig. 9, Supplementary Material online) (DeGiorgio et al., 2014; Siewert and Voight, 2017). This is because a higher mutation rate results in more SNPs fixing in allelic class, whereas a lower mutation rate results in less recombination decoupling SNPs from the balanced SNP. We found that power decreases with increasing population size (supplementary fig. 10, Supplementary Material online). This is expected because a higher population size increases the expected TMRCA at a neutral locus, resulting in a smaller difference in TMRCA between balanced and neutral loci.

We next scan the human genome for evidence of balancing selection, applying the $\beta_{\text{std}}^{\left(2\right)}$ method (supplementary information, Supplementary Material online) to the YRI, CEU, and CHB populations from the 1000 genomes project (The 1000 Genomes Consortium, 2015) (supplementary fig. 11, Supplementary Material online). To prepare files for input into BetaScan we used the glactools toolkit, which can output in BetaScan format with the option to include substitutions (Renaud, 2018) (supplementary information, Supplementary Material online). As expected, there is a high correlation between the three *β* statistics, the unstandardized and standardized statistics (supplementary tables 1 and 2, Supplementary Material online), and scores in the three populations (supplementary tables 3 and 4, Supplementary Material online).

We next tested if $\beta^{\left(2\right)}$ captures genomic regions which are likely true positives. Trans-species haplotypes between human and chimpanzee are an independent measure of balancing selection and perhaps the closest available thing to a set of true positive loci under long-term balancing selection. We found that SNPs in trans-species haplotypes from Leffler et al. (2013) had a significantly higher $\beta_{\text{std}}^{\left(2\right)}$ value than SNPs which are not (Mann–Whitney *U P*-value =  $1.08\times 10^{-14}$). We also compared the values of $\beta_{\text{std}}^{\left(2\right)}$ versus T2 in SNPs in trans-haplotypes. We found that after removing rare variants, the mean percentile for SNPs in these haplotypes was 0.63 for $\beta_{\text{std}}^{\left(2\right)}$ and 0.66 for T2. Before removing rare variants, the mean percentile for $\beta_{\text{std}}^{\left(2\right)}$ was 0.64 and T2 was 0.72. This indicates that $\beta_{\text{std}}^{\left(2\right)}$ may have additional noise when calculated on rare variants in real data due to demography that is not captured in the theoretical site frequency spectrum. These effects will be captured in the empirical neutral distribution used by *T*2, increasing its power. As with the $\beta^{\left(1\right)}$ statistics, we therefore recommend only applying $\beta^{\left(2\right)}$ to SNPs which are not rare. Because balanced variants are unlikely to be maintained at extreme equilibrium frequencies (Ewens and Thomson, 1970; Nei and Roychoudhury, 1973), this should not hurt power.

If $\beta_{\text{std}}^{\left(2\right)}$ is capturing true balanced loci, we would expect scores to be higher in regions with a demonstrated functional importance, such as eQTLS or GWAS SNPs. To test this hypothesis, we overlapped our top SNPs with the GWAS catalog (Buniello et al., 2019) (supplementary information, Supplementary Material online), and significant eQTLs from the GTeX project (The GTEx Consortium, 2015). Using logistic regression, we found that both standardized and unstandardized $\beta^{\left(2\right)}$ were positively predictive of a SNP being an eQTL (supplementary table 5, Supplementary Material online) or in the GWAS catalog (supplementary table 6, Supplementary Material online). This remained true after taking into account potential confounding factors such as minor allele frequency and distance to the nearest gene. Reassuringly, included in our top hits are previously discovered loci, including several loci in the HLA region (supplementary fig. 11, Supplementary Material online).

**Fig. 2.**
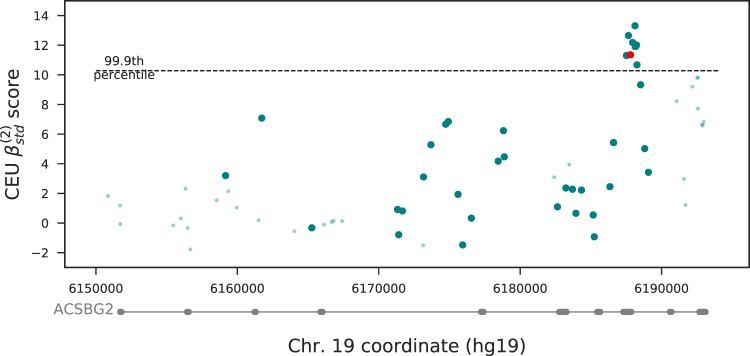
—The ACSBG2 locus shows evidence of balancing selection in the CEU population. The red dot indicates two missense mutations. The large circles indicate SNPs at the frequency of the putatively balanced haplotype, whereas the small circles indicate SNPs at other frequencies. Only SNPs passing our filtering for quality are plotted.

Two missense SNPs in the gene *ACSBG2*, rs17856650 and rs17856651, have standardized *β* scores in the top 99.9 percentile in the CEU population (fig. 2), top 99.5 percentile in the CHB population and top 99 percentile in the YRI population. Confirming this finding, this gene was one of the top 100 loci detected using the *T*2 statistic (DeGiorgio et al., 2014), but has been previously uncharacterized. In all three populations, the percentile of the standardized $\beta^{\left(2\right)}$ score is slightly higher than the unstandardized, demonstrating the power of standardization in regions with a lower mutation rate, such as within genes. The Acyl-CoA Synthetase Bubblegum Family Member 2 (ACSBG2) gene is involved in fatty acid metabolism (Pei et al., 2006). ACSBG2 may play a role in spermatogenesis (Fraisl et al., 2006), a process that has been previously associated with balancing selection (Bitarello et al., 2018). In addition, this gene was highlighted as harboring potentially deleterious lineage-specific nonsynonymous single nucleotide changes in bonobo (Han et al., 2019). However, the reported bonobo changes are at a different location in the gene than the two missense SNPs in humans.

The results presented in this manuscript demonstrate the power and wide-applicability of the BetaScan2 suite of statistics. In addition to the ability to standardize *β* and the implementation of $\beta^{\left(2\right)}$, BetaScan2 can calculate *β*_std_ on a predefined window of interest, instead of requiring the use of a sliding window across the genome. This locus-based calculation is made possible by standardization allowing comparison of scores across different window sizes, and enables the use of *β* on species for which only a small fraction of the genome is available. BetaScan2 is freely available at https://github.com/ksiewert/BetaScan.

## Materials and Methods

We first derive a closed-form solution for the expected number of substitutions under the neutral model (supplementary information, Supplementary Material online). This expression leads to an estimator of the mutation rate ${\hat{\theta }}_{D}$ based upon the number of substitutions *D*:

(1) $${{\hat{\theta }}_{D}}_{}=\frac{D}{T/(2N_{e})+1/n}.$$

Here *T* is the divergence time between the two species, *N*_e_ is the effective population size, and *n* is the number of surveyed chromosomes. To incorporate information from substitutions, we replace ${\hat{\theta }}_{W}$ from the original unfolded statistic with ${\hat{\theta }}_{D}$ to define $\beta^{\left(2\right)}$:

(2) $$\beta^{\left(2\right)}={\hat{\theta }}_{\beta }-{\hat{\theta }}_{D}.$$

Under long-term balancing selection, variants nearby the selected site are maintained at similar allele frequencies rather than fixing in the population, resulting in an increased estimate of $\theta_{\beta }$ and reduced estimate of $\theta_{D}$. Therefore, *β* is expected to score above zero in the presence of long-term balancing selection, whereas it will be below or around zero under neutrality.

Intuitively, the variances of the *β* statistics depend on the population size, survey sample size, equilibrium frequency, and mutation rate. We derive the variances of $\beta^{(1)*}$ and $\beta^{\left(2\right)}$ (supplementary material, Supplementary Material online), allowing for comparison of scores across samples, window sizes and genomic loci where these factors may differ. Our expressions for variance match simulation results (supplementary figs. 1 and 2, Supplementary Material online). To obtain the variance of $\beta^{\left(1\right)}$ we used the framework reported in Achaz (2009) (supplementary information, Supplementary Material online). We note that the expected value of all *β* statistics is zero. This leads to, for $\beta^{\left(2\right)}$ for instance:

(3) $$\beta_{\text{std}}^{\left(2\right)}=\frac{\beta^{\left(2\right)}}{\sqrt{\text{Var}[\beta^{\left(2\right)}]}}=\frac{{\hat{\theta }}_{\beta }-{\hat{\theta }}_{D}}{\sqrt{\text{Var}[{\hat{\theta }}_{\beta }]+\text{Var}[{\hat{\theta }}_{D}]}}.$$

Full derivations for $\beta_{\text{std}}^{\left(1\right)},\;\beta_{\text{std}}^{(1)*}$, and $\beta_{\text{std}}^{\left(2\right)}$ can be found in the supplementary information, Supplementary Material online. Calculating the variance of all three *β* statistics requires an estimate of $\hat{\theta }$, the underlying mutation rate. The variance of $\beta^{\left(2\right)}$ also requires an estimate of the speciation time. We discuss techniques for estimation of these parameters in the supplementary information, Supplementary Material online. However, the *β* statistics are robust to some specification error (supplementary figs. 3 and 4, Supplementary Material online). We recommend $\beta_{\text{std}}^{(1)*}$ in cases without outgroup data to polarize ancestral states, $\beta_{\text{std}}^{(1)*}$ when ancestral states are known but no informative outgroup is available to call substitutions, and $\beta_{\text{std}}^{\left(2\right)}$ when substitutions are available.

## Supplementary Material

evaa013_Supplementary_Data
