# Supplementary material for: BetaScan2: Standardized Statistics to Detect Balancing Selection Utilizing Substitution Data
Source: Genome Biol Evol. 2020 Feb 3;12(2):3873–7. doi: 10.1093/gbe/evaa013 (PMC7058154; doi:10.1093/gbe/evaa013)
Supplement: evaa013_Supplementary_Data [file evaa013_supplementary_data.pdf]

# Supplementary Information: BetaScan2: Standardized statistics to detect balancing selection utilizing substitution data

Katherine M. Siewert, Benjamin F. Voight

## Contents

**1 Supplementary figures** **2**

**2 Supplementary tables** **10**

**3 Derivation of $\hat{\theta}_D$ and its variance** **12**

3.1 Derivations of $\theta_D^{Anc}$ . . . . . . . . . . 14

**4 Variance of the folded $\beta$ statistic** **15**

**5 Standardized $\beta$ statistics** **16**

**6 Estimation of the background mutation rate and speciation time** **16**

**7 Method of power analysis** **17**

**8 Discussion of power analysis results** **18**

**9 Application to human** **19**

# 1 Supplementary figures

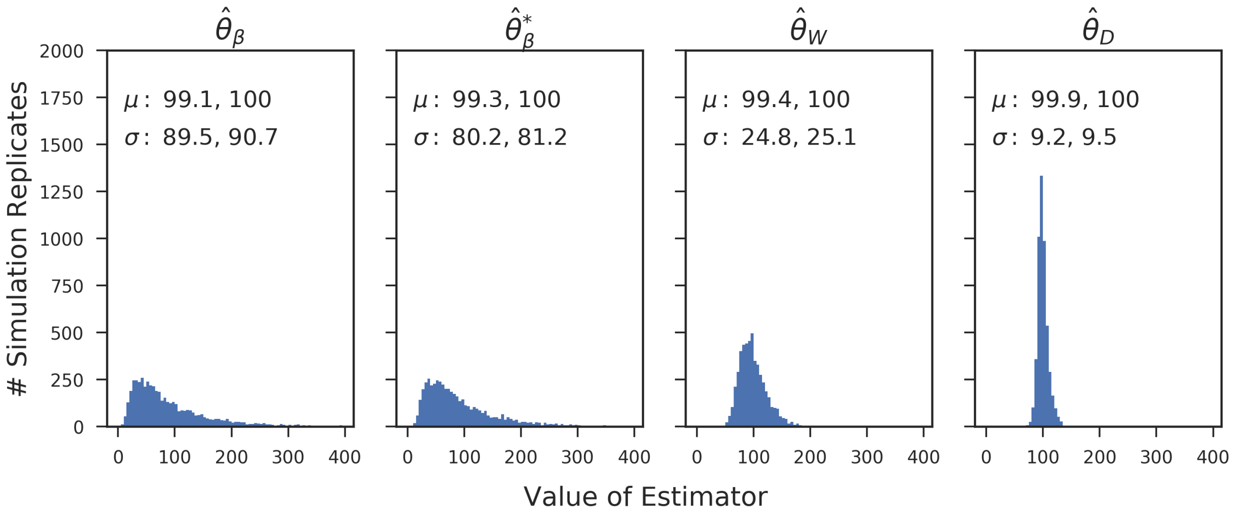


Figure 1: Distribution of each $\theta$ estimator on simulated 100kb windows with no selection or recombination and equilibrium demography. Core frequencies were chosen to be 0.5, regardless of whether a SNP of that frequency was found in the window. Mean ($\mu$) and standard deviation ($\sigma$) are displayed, with the first number being the sample value, and the second being the theoretical value.

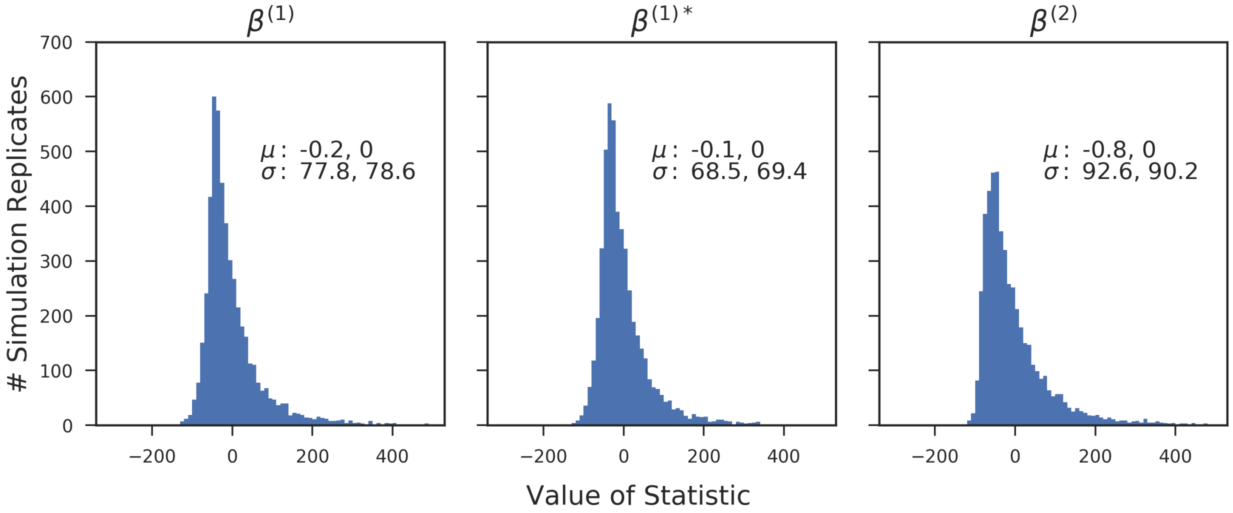


Figure 2: Distribution of each $\beta$ statistic on simulated 100kb windows with no selection or recombination and equilibrium demography. Core frequencies were chosen to be 0.5, regardless of whether a SNP of that frequency was found in the window. Mean ($\mu$) and standard deviation ($\sigma$) are displayed, with the first number being the sample value, and the second being the theoretical value.

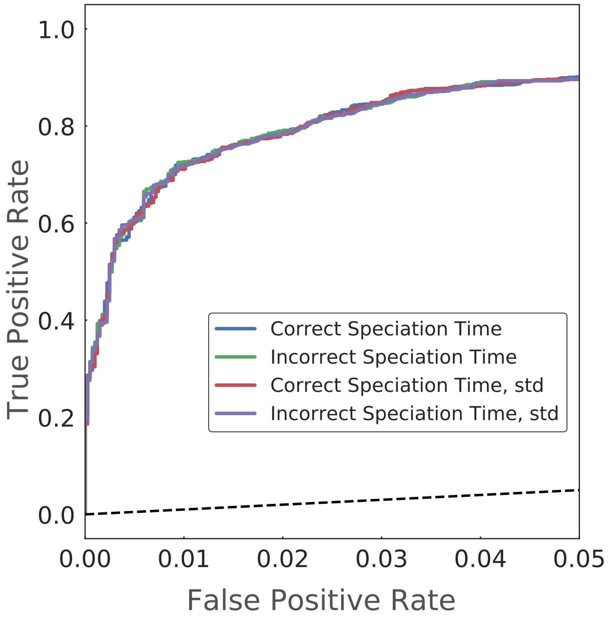


Figure 3: Power of $\beta^{(2)}$ and $\beta^{(2)}_{std}$ when the speciation time parameter is correctly specified as 250,000 generations prior to sampling versus when it is underestimated by 100,000 generations. An equilibrium frequency of 50% and a selection age of 250,000 generations prior to sampling were used.

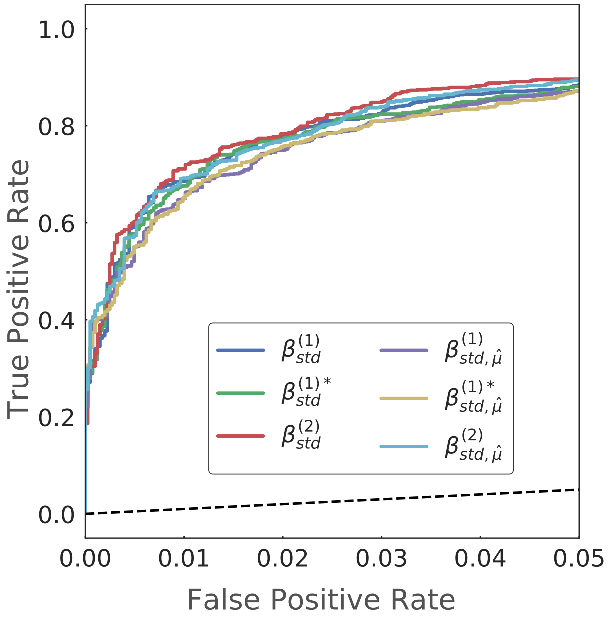


Figure 4: Power of $\beta$ statistics when the background mutation rate is estimated using a 10kb window centered at the core SNP ($\hat{\mu}$) versus using the true mutation rate. A mutation rate of $2.5 \times 10^{-8}$, an equilibrium frequency of 50% and a selection age of 250,000 generations prior to sampling was used.

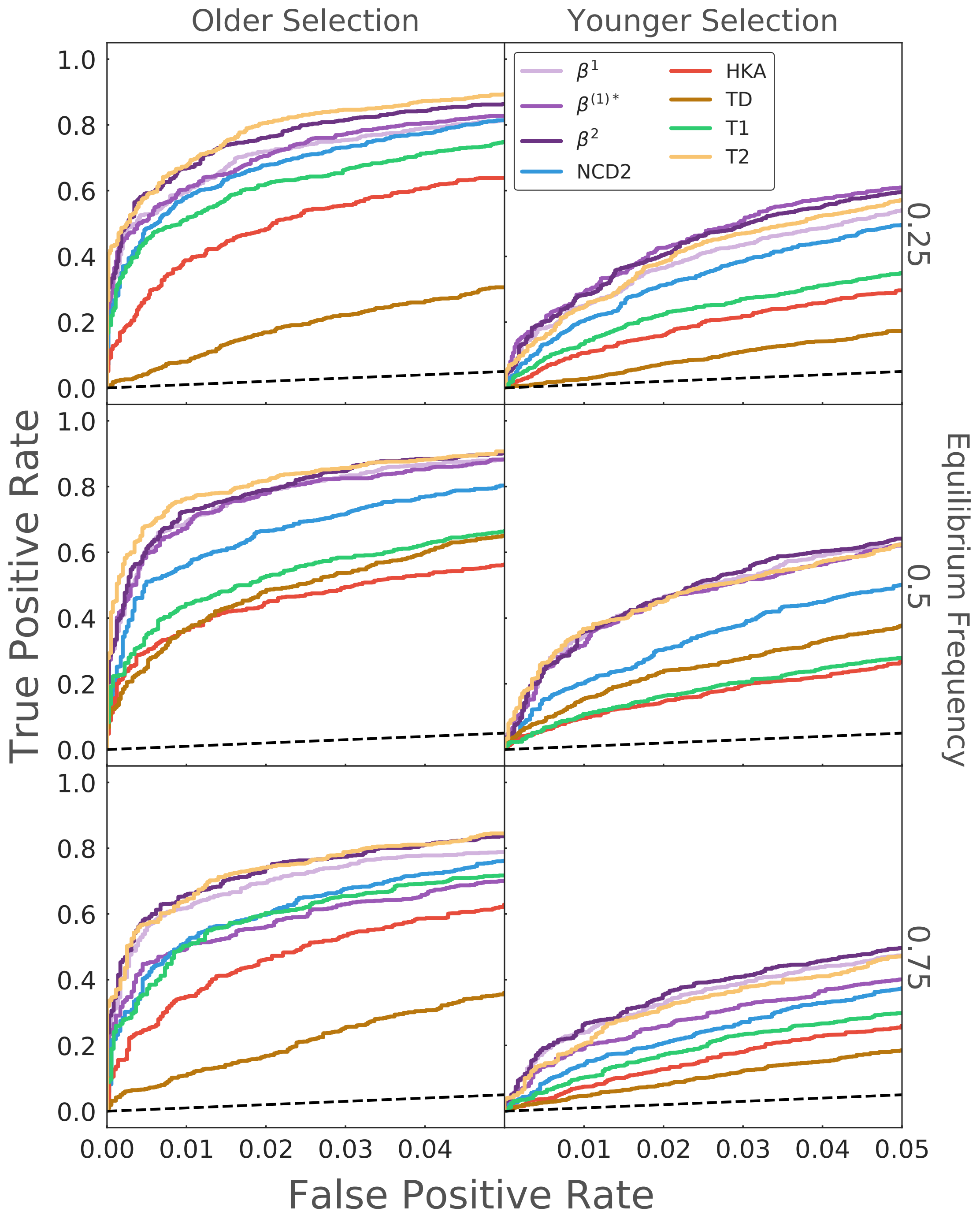


Figure 5: Power of methods to detect long-term balancing selection. Power was calculated based on simulation replicates containing only neutral variants (True Negatives) or containing a balanced variant that was introduced (True Positives). The score of the balanced SNP was used for each statistic, as was the score of a SNP from the neutral simulations matched for frequency. Rows correspond to simulations of balanced alleles at equilibrium frequencies 0.25, 0.50, and 0.75. Columns correspond to older and more recent selection, beginning 250,000 and 100,000 generations prior to sampling, respectively. The black line goes from the origin to a true and false positive rate of one, and would correspond to a method with no discriminatory power.

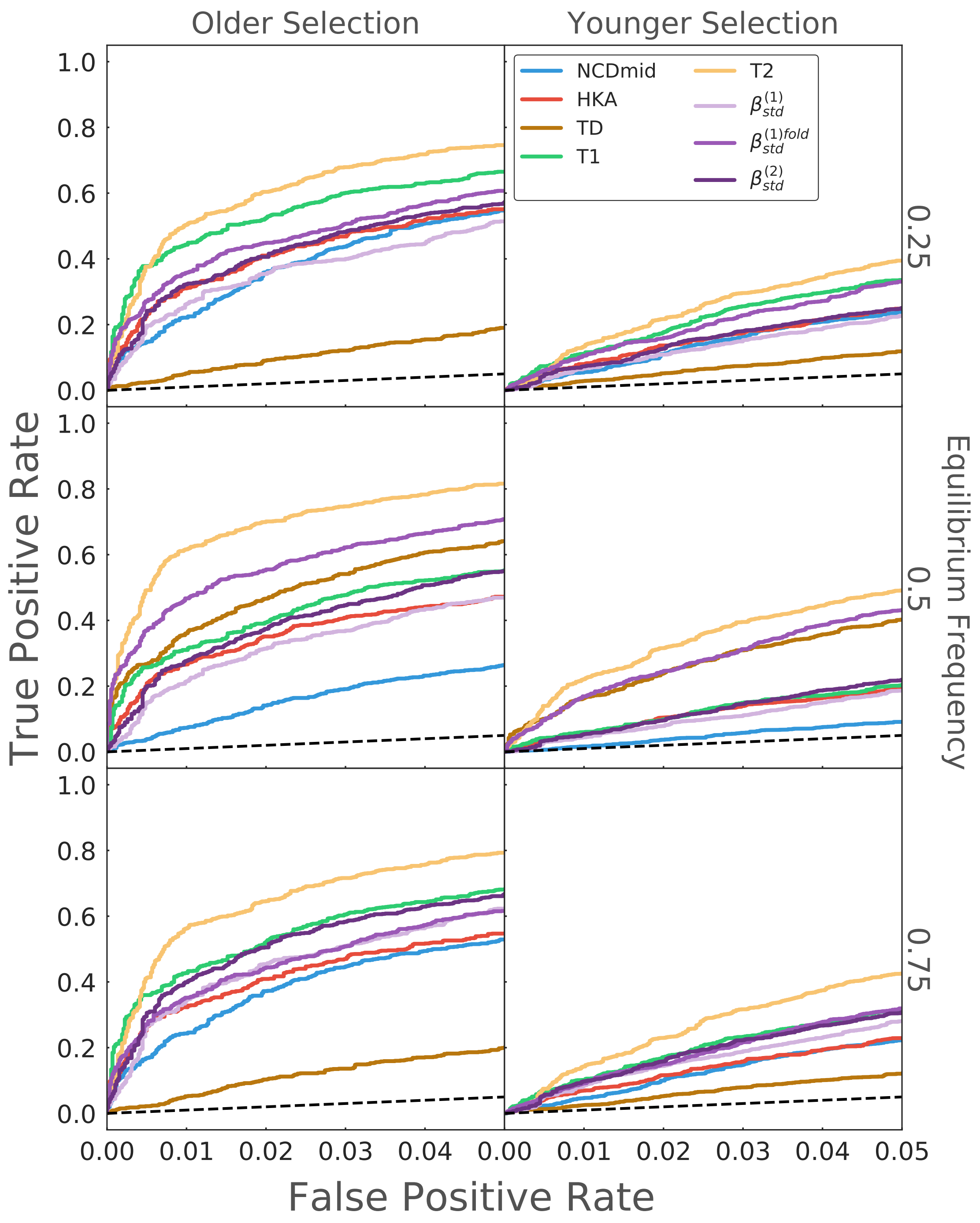


Figure 6: Power of methods to detect ancient balancing selection without matching for allele frequency. Power was calculated based on simulation replicates containing only neutral variants (True Negatives) or containing a balanced variant that was introduced (True Positives). The maximum value of each statistic in each simulated 10kb window was used. Rows correspond to simulations of balanced alleles at equilibrium frequencies 0.25, 0.50, and 0.75. Columns correspond to older and more recent selection, beginning 250,000 and 100,000 generations prior to sampling, respectively.

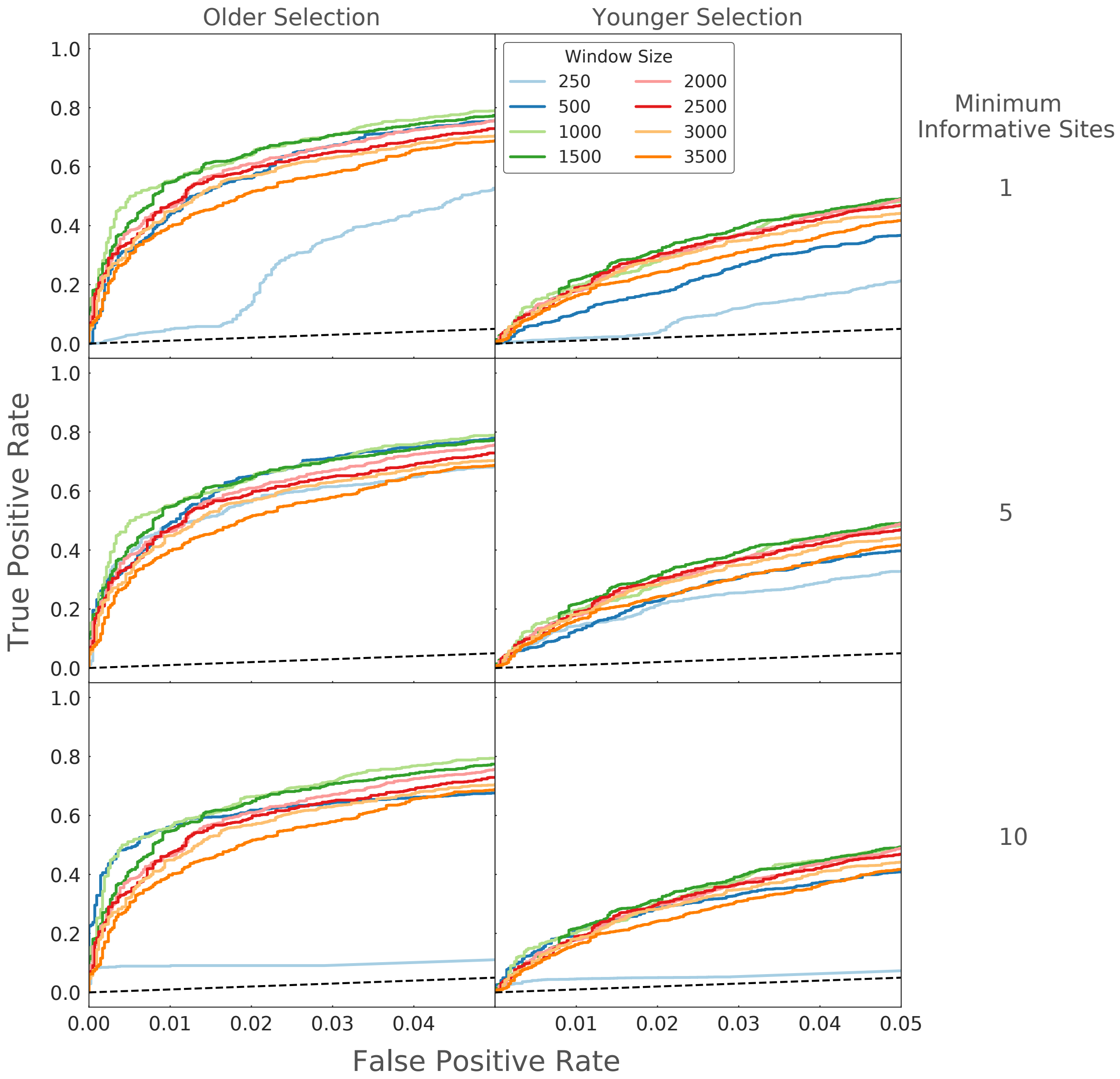


Figure 7: Power of the $NCD2$ statistic using different window sizes and minimum number of informative sites (SNPs plus substitutions). Here, we show that the 1kb window we used for $NCD2$ is optimal for power. In addition, Bitarello *et al.* (2018) suggested that $NCD2$ may require a minimum of 5 or 10 informative sites for maximum power. We show that when using an optimal window size, no minimum is needed under our simulation parameters. For each row, any windows with less than the given number of informative sites were called as neutral. Units are in base pairs. An equilibrium frequency of 50% was used. Columns correspond to older and more recent selection, beginning 250,000 and 100,000 generations prior to sampling, respectively. Window size analysis of other methods can be found in the supplement of Siewert and Voight (2017).

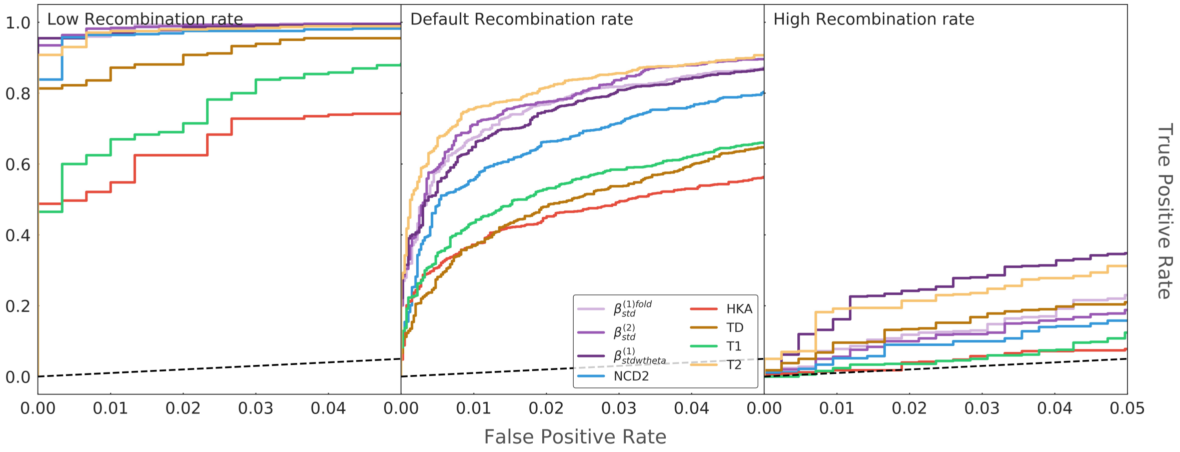


Figure 8: Power of statistics to detect balancing selection in simulations with different recombination rates: low $(2.5x10^{-9})$, default $(2.5x10^{-8})$ and high $(2.5x10^{-9})$. The balanced SNP is of equilibrium frequency 50% and 250,000 generations old. We were unable to calculate $T1$ or $T2$ on the alternative population sizes, because the grids of simulations required for these statistic under alternative demographic parameters are unavailable with the BALLET software implementing these statistics.

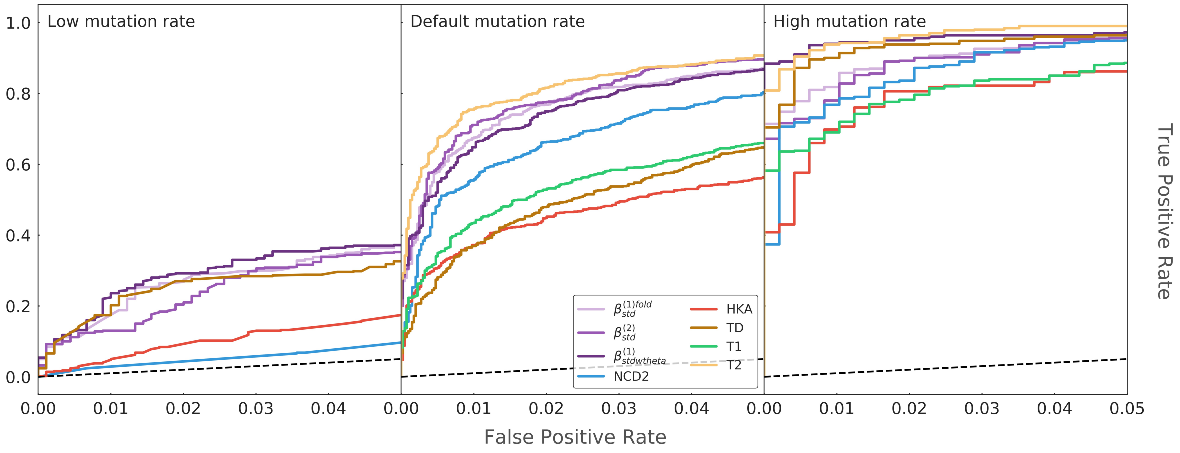


Figure 9: Power of statistics to detect balancing selection in simulations with different mutation rates: low $(2.5x10^{-9})$, default $(2.5x10^{-8})$ and high $(2.5x10^{-9})$. The balanced SNP is of equilibrium frequency 50% and 250,000 generations old. $T1$ and $T2$ were not calculated for the low mutation rate simulations because there were not enough SNPs in the 10kb window for which to run the scan.

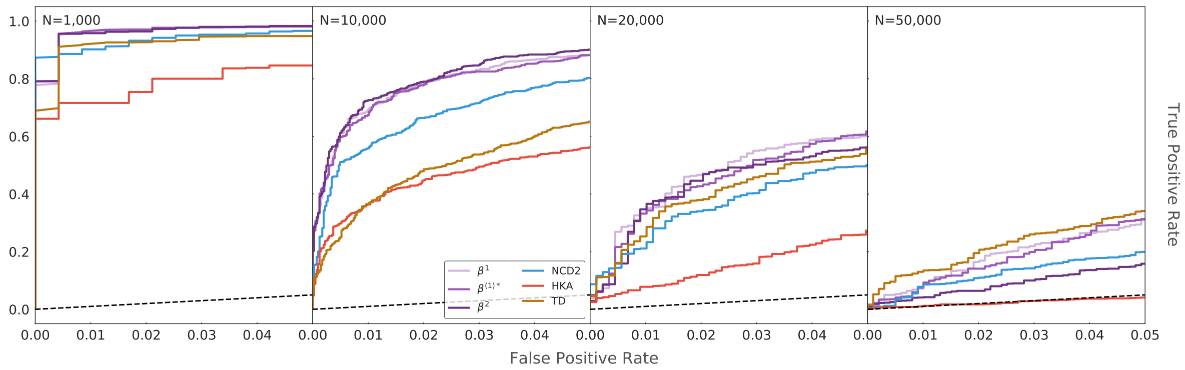


Figure 10: Power of statistics to detect balancing selection in simulations with different effective population sizes. The balanced SNP is of equilibrium frequency 50% and 250,000 generations old.

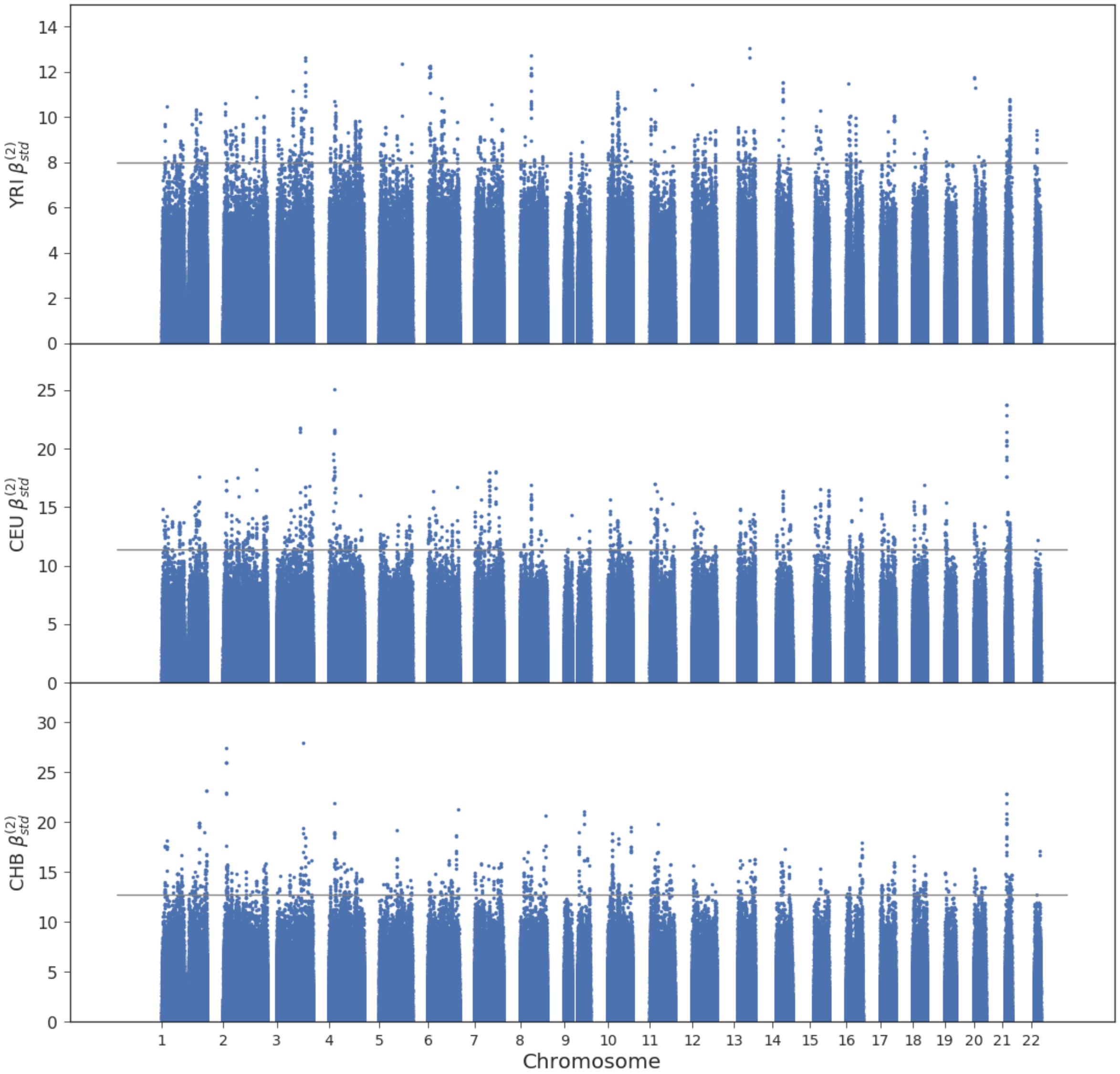


Figure 11: $\beta_{std}^{(2)}$ from 3 different 1000 Genomes population groups. Grey line denotes the top 99.95 percentile of scores in that population. Only SNPs of minor allele frequency of at least 5% in all population and which pass the sequence quality mask are included.

## 2 Supplementary tables

| | $\beta^{(1)}$ | $\beta^{(1)}_{std}$ | $\beta^{(1*)}$ | $\beta^{(1*)}_{std}$ | $\beta^{(2)}$ | $\beta^{(2)}_{std}$ |
|---|---|---|---|---|---|---|
| $\beta^{(1)}$ | 1.000000 | 0.950280 | 0.532831 | 0.499866 | 0.941937 | 0.887018 |
| $\beta^{(1)}_{std}$ | | 1.000000 | 0.474108 | 0.488001 | 0.877132 | 0.917376 |
| $\beta^{(1*)}$ | | | 1.000000 | 0.897500 | 0.513515 | 0.452262 |
| $\beta^{(1*)}_{std}$ | | | 0.897500 | 1.000000 | 0.466833 | 0.457908 |
| $\beta^{(2)}$ | | | | | 1.000000 | 0.942435 |
| $\beta^{(2)}_{std}$ | | | | | | 1.000000 |

Table 1: Pearson's correlation coefficient between the 6 different $\beta$ statistics in the YRI population. Only SNPs passing the variant quality masks were included.

| | $\beta^{(1)}$ | $\beta^{(1)}_{std}$ | $\beta^{(1*)}$ | $\beta^{(1*)}_{std}$ | $\beta^{(2)}$ | $\beta^{(2)}_{std}$ |
|---|---|---|---|---|---|---|
| $\beta^{(1)}$ | 1.000000 | 0.954548 | 0.909405 | 0.891970 | 0.952238 | 0.895653 |
| $\beta^{(1)}_{std}$ | | 1.000000 | 0.859370 | 0.918742 | 0.896334 | 0.925701 |
| $\beta^{(1*)}$ | | | 1.000000 | 0.946173 | 0.868173 | 0.808714 |
| $\beta^{(1*)}_{std}$ | | | | 1.000000 | 0.840925 | 0.854370 |
| $\beta^{(2)}$ | | | | | 1.000000 | 0.947561 |
| $\beta^{(2)}_{std}$ | | | | | | 1.000000 |

Table 2: Pearson's correlation coefficient between the 6 different $\beta$ statistics in the YRI population. Only SNPs passing the variant quality masks and of minor allele frequency of at least 15% were included.

| | YRI $\beta^{(2)}$ | YRI$\beta^{(2)}_{std}$ | CEU $\beta^{(2)}$ | CEU$\beta^{(2)}_{std}$ | CHB $\beta^{(2)}$ | CHB$\beta^{(2)}_{std}$ |
|---|---|---|---|---|---|---|
| YRI $\beta^{(2)}$ | 1.000000 | 0.941067 | 0.811245 | 0.704202 | 0.795841 | 0.675529 |
| YRI$\beta^{(2)}_{std}$ | | 1.000000 | 0.747083 | 0.734074 | 0.732944 | 0.705801 |
| CEU $\beta^{(2)}$ | | | 1.000000 | 0.919521 | 0.884536 | 0.784504 |
| CEU$\beta^{(2)}_{std}$ | | | | 1.000000 | 0.799528 | 0.831685 |
| CHB $\beta^{(2)}$ | | | | | 1.000000 | 0.913974 |
| CHB$\beta^{(2)}_{std}$ | | | | | | 1.000000 |

Table 3: Pearson's correlation coefficient between the unstandardized and standardized $\beta^{(2)}$ statistics in 3 populations. Only scores of SNPs present in all 3 population were used. Only SNPs passing the variant quality masks were included.

| | YRI $\beta^{(2)}$ | YRI$\beta^{(2)}_{std}$ | CEU $\beta^{(2)}$ | CEU$\beta^{(2)}_{std}$ | CHB $\beta^{(2)}$ | CHB$\beta^{(2)}_{std}$ |
|---|---|---|---|---|---|---|
| YRI $\beta^{(2)}$ | 1.000000 | 0.942745 | 0.826468 | 0.721393 | 0.819100 | 0.705528 |
| YRI$\beta^{(2)}_{std}$ | | 1.000000 | 0.764007 | 0.753123 | 0.755459 | 0.737578 |
| CEU $\beta^{(2)}$ | | | 1.000000 | 0.920308 | 0.901193 | 0.808905 |
| CEU$\beta^{(2)}_{std}$ | | | | 1.000000 | 0.814493 | 0.858317 |
| CHB $\beta^{(2)}$ | | | | | 1.000000 | 0.914297 |
| CHB$\beta^{(2)}_{std}$ | | | | | | 1.000000 |

Table 4: Pearson's correlation coefficient between the unstandardized and standardized $\beta^{(2)}$ statistics in 3 populations. Only scores of SNPs present in all 3 population were used. Only SNPs passing the variant quality masks and of minor allele frequency of at least 15% were included.

| Feature | coefficient | z-score | p-value |
|---|---|---|---|
| intercept | -3.3781 | -1621.759 | <2.23x10$^{-308}$ |
| $\beta^{(2)_{std}}$ | 0.0490 | 63.262 | <2.23x10$^{-308}$ |
| Distance to nearest gene | -5.871e-06 | -502.175 | <2.23x10$^{-308}$ |
| Distance to nearest gene squared | 2.555e-12 | 327.349 | <2.23x10$^{-308}$ |
| MAF | 20.2278 | 887.367 | <2.23x10$^{-308}$ |
| MAF squared | -29.8492 | -29.752 | 3.20 x 10$^{-193}$ |

Table 5: Multivariable logistic regression of a SNP or any of its proxies being an eQTL in any tissue in GTExv7 regressed against several features, including $\beta^{(2)}_{std}$ score in YRI.

| Feature | coefficient | z-score | p-value |
|---|---|---|---|
| intercept | -5.7559 | -950.179 | <2.23x10$^{-308}$ |
| $\beta^{(2)_{std}}$ | 0.1127 | 62.142 | <2.23x10$^{-308}$ |
| Distance to nearest gene | -2.072e-06 | -89.393 | <2.23x10$^{-308}$ |
| Distance to nearest gene squared | 9.227e-13 | 56.147 | <2.23x10$^{-308}$ |
| MAF | 16.8289 | 282.689 | <2.23x10$^{-308}$ |
| MAF squared | -24.4103 | -193.243 | <2.23x10$^{-308}$ |

Table 6: Multivariable logistic regression of a SNP or its proxies being in the GWAS catalog regressed against several features, including $\beta^{(2)}_{std}$ score in YRI.

# 3 Derivation of $\hat{\theta}_D$ and its variance

We first derive our estimator of the mutation rate based on the divergence between two species, $\hat{\theta}_D$. To measure divergence, we use the number of substitutions, which we define as the nucleotide positions in which the outgroup individual is different than all ingroup individuals. We note that this differs from the measure of between-species divergence in the HKA test, which is instead the average number of differences between a randomly selected ingroup and outgroup gamete (Hudson *et al.*, 1987). We choose our measure of divergence, because as noted in Hudson *et al.* (1987), it has slightly lower variance. We assume that there is a single outgroup individual and that the time since speciation is sufficiently long that the ingroup coalescence occurred prior to coalescing with the outgroup. Throughout our derivations, we assume Hardy-Weinberg equilibrium, an infinite sites model, and no recombination. In practice, recombination will act to decrease the variance, making our tests conservative (Tajima, 1989).

We model divergence using the coalescence tree of the ingroup individuals and the outgroup individuals. This tree contains two parts that can contribute to substitutions. Considering the tree backward in time, these parts are (i) after the coalescence of the common ancestor of the ingroup and outgroup individuals and (ii) after the coalescence within each species, but before coalescence between the two species.

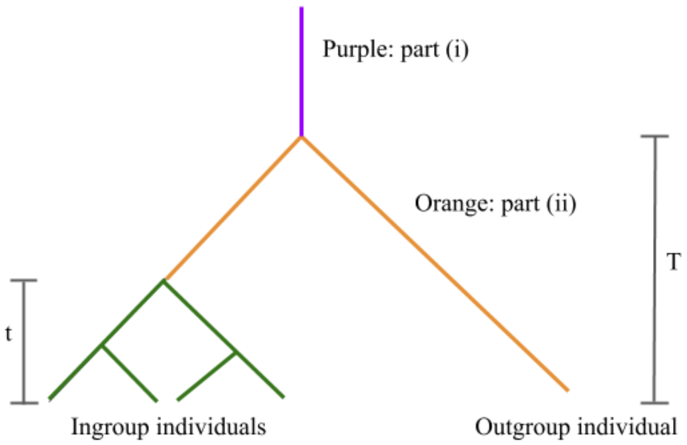


**Expected number and variance of substitutions from part (ii),** $D_{(ii)}$:
The number of substitutions in part (ii) of the tree is Poisson distributed, $D_{(ii)} \sim Poisson(\mu L)$, where $\mu$ is the mutation rate and $L$ is the branch length in part (ii). The branch length is given by $L \approx 2T - t$, where $T$ is the coalescence time of the ingroup and outgroup (i.e. speciation time), and $t$ is the coalescence time of the ingroup species. The expected value and variance of $t$ is given in Tavaré (1984). Let $F$ be the Poisson distributed variable representing the number of mutations along these branches, and $G$ represent the height of the ingroup coalescence tree. Define the surveyed size of sampled chromosomes to be $n$, the effective population size as $N_e$, and $\theta = 4N_e\mu$ with $\mu$ as the usual mutation rate per base per generation. We can derive the expected value and variance of $D_{(ii)}$ using the properties of compound probability distributions, the theorem for moments of the height of a coalescent tree from Tavaré (1984) and the mean and variance of the Poisson distribution. First, we find the expected value:

$$\begin{aligned}
E[D_{(ii)}] &= E_G[E_F[D_{(ii)}|L]] \\
&= E_G[\mu(2T - t)] \\
&= 2T\mu - E_G[t]\mu \\
&= 2T\mu - 4N_e\big(1 - \frac{1}{n}\big)\mu \\
&= \theta\Big(\frac{T}{2N_e} - (1 - \frac{1}{n})\Big)
\end{aligned} \tag{1}$$

Next, we find the variance of $D_{(ii)}$, using the variance of $t$ from Tavaré (1984):

$$\begin{aligned}
Var[D_{(ii)}] &= E_G[Var_F[D_{(ii)}|L]] + Var_G[E_F[D_{(ii)}|L]] \\
&= E_G[(2T - t)\mu] + Var_G[(2T - t)\mu] \\
&= 2T\mu - E_G[t]\mu + Var_G[2T\mu - t\mu] \\
&= 2T\mu - 4N_e(1 - \frac{1}{n})\mu + \mu^2 Var_G[t] \\
&= 2T\mu - 4N_e\mu + \frac{4N_e\mu}{n} + (4N_e\mu)^2\Big(\sum_{i=2}^{n}\frac{1}{i^2(i-1)^2}\Big)
\end{aligned} \tag{2}$$

**Expected number and variance of substitutions from part (i), $D_{(i)}$:**
We obtain the expected number and variance of $D_{(i)}$ by noting that it is equivalent to the difference between two random gametes from Watterson (1975).

$$E[D_{(i)}] = \theta \tag{3}$$

$$Var[D_{(i)}] = \theta + \theta^2 \tag{4}$$

**Expected number and variance of substitutions from whole tree:**
We denote the total number of substitutions as $D = D_{(i)} + D_{(ii)}$. From Eqs. (1) and (3) above, the expected value is then given by:

$$\begin{aligned}
E[D] &= E[D_{(i)}] + E[D_{(ii)}] \\
&= \theta + \theta\Big(\frac{T}{2N_e} - (1 - \frac{1}{n})\Big) \\
&= \theta\Big(\frac{T}{2N_e} + \frac{1}{n}\Big)
\end{aligned} \tag{5}$$

Because the coalescent process in part (i) is independent of the coalescence process in part (ii), we can simply add variances from Eqs. (2) and (4) above to obtain:

$$\begin{aligned}
Var[D] &= Var[D_i] + Var[D_{ii}] \\
&= \theta + \theta^2 + 2T\mu - 4N_e\mu + \frac{4N_e\mu}{n} + (4N_e\mu)^2(\sum_{i=2}^{n}\frac{1}{i^2(i-1)^2}) \\
&= \theta^2 + \frac{T\theta}{2N_e} + \frac{\theta}{n} + \theta^2\sum_{i=2}^{n}\frac{1}{i^2(i-1)^2}
\end{aligned} \tag{6}$$

We note that our results for the mean and variance of $D$ are simplified forms of equations 29C and 31C from Hey (1991) when taking the large $T$ limit.

Solving for $\theta$ in Eq. 5 we obtain $\hat{\theta}_D$:

$$\hat{\theta}_D = \frac{D}{\frac{T}{2N_e} + \frac{1}{n}} \tag{7}$$

The variance of $\hat{\theta}_D$ is then:

$$
\begin{aligned}
Var[\hat{\theta}_D] &= Var\left[\frac{D}{\frac{T}{2N_e}+\frac{1}{n}}\right] \\
&= \left(\frac{1}{\frac{T}{2N_e}+\frac{1}{n}}\right)^2 Var[D] \\
&= \left(\frac{1}{\frac{T}{2N_e}+\frac{1}{n}}\right)^2 \left(\theta^2 + \frac{T\theta}{2N_e} + \frac{\theta}{n} + \theta^2 \sum_{i=2}^{n} \frac{1}{i^2(i-1)^2}\right)
\end{aligned} \tag{8}
$$

## 3.1 Derivations of $\theta_D^{Anc}$

We now derive $\hat{\theta}_D^{Anc}$, which is an estimate of the mutation rate based only on substitutions that occurred in the ingroup lineage after the speciation event. This can be used in place of the standard $\hat{\theta}_D$ if the root allele can be inferred through the use of multiple outgroup species.

$$
E[D^{Anc}] = T\mu - E_G[t]\mu \tag{9}
$$
$$
= \mu(T - E_G[t]) \tag{10}
$$
$$
= \theta\left(\frac{T}{4N_e} - (1 - \frac{1}{n})\right) \tag{11}
$$

Therefore,

$$
\hat{\theta}_D^{Anc} = \frac{D}{\frac{T}{4N_e} - (1-\frac{1}{n})}
$$

Now for the variance:

$$
\begin{aligned}
Var[D^{Anc}] &= E_G[Var_F[D_{(Anc)}|L]] + Var_G[E_F[D_{(Anc)}|L]] \\
&= E_G[\mu(T-t)] + Var_G[\mu(T-t)] \\
&= \mu T - 4N_e(1-\frac{1}{n})\mu + \mu^2 Var_G[t] \\
&= \frac{T\theta}{4N_e} - \theta + \frac{\theta}{n} + \theta^2\left(\sum_{i=2}^{n} \frac{1}{i^2(i-1)^2}\right)
\end{aligned}
$$

$$
\begin{aligned}
Var[\hat{\theta}_D^{Anc}] &= Var\left[\frac{D^{Anc}}{\frac{T}{2N_e}+\frac{1}{n}}\right] \\
&= \left(\frac{1}{\frac{T}{2N_e}+\frac{1}{n}}\right)^2 Var[D^{Anc}] \\
&= \left(\frac{1}{\frac{T}{2N_e}+\frac{1}{n}}\right)^2 \left(\frac{T\theta}{4N_e} - \theta + \frac{\theta}{n} + \theta^2\left(\sum_{i=2}^{n} \frac{1}{i^2(i-1)^2}\right)\right)
\end{aligned}
$$

# 4 Variance of the folded $\beta$ statistic

The formulation for $\beta^{1(*)}$ does not fall into the class of neutrality tests based on the folded site frequency spectrum studied in Achaz (2009), because the folded frequency of each SNP is not considered in our formulation. Therefore, we provide a derivation below. $\phi$ and $\rho$ are defined in Achaz (2009) and $d_i$ is the measure of frequency similarity from Siewert and Voight (2017). $S_g(i)$ is the number of SNPs in the window of folded frequency g(i) and is analogous to $\eta_i$ in Achaz (2009). Set $m = \lceil \frac{n}{2} \rceil$, where $\lceil\rceil$ denotes the ceiling. We refer to the estimator of $\hat{\theta}_\beta^{fold}$ reported in Siewert and Voight (2017) as $\hat{\theta}_\beta^*$. The variance of $\beta^{(1)*}$ is:

$$Var[\hat{\theta}_\beta^* - \hat{\theta}_W] = Var[\hat{\theta}_\beta^*] + Var[\hat{\theta}_W] - 2Cov[\hat{\theta}_\beta^*, \hat{\theta}_W] \tag{12}$$

First, we derive the variance of $\hat{\theta}_\beta^*$ to be:

$$\begin{aligned}
Var[\hat{\theta}_\beta^*] &= Var\left[\frac{\sum_{i=1}^m d_i S_{g(i)}}{\sum_{i=1}^m d_i(\frac{1}{i} + \frac{1}{n-i})\frac{1}{1+\delta_{i,n-i}}}\right] \\
&= \left(\sum_{i=1}^m d_i\Big(\frac{1}{i} + \frac{1}{n-i}\Big)\frac{1}{1+\delta_{i,n-i}}\right)^{-2} Var\left[\sum_{i=1}^m d_i S_{g(i)}\right] \\
&= \left(\sum_{i=1}^m d_i\Big(\frac{1}{i} + \frac{1}{n-i}\Big)\frac{1}{1+\delta_{i,n-i}}\right)^{-2} \left(\sum_{i=1}^m Var[d_i S_{g(i)}] + \sum_{i\neq j} Cov[d_i S_{g(i)} d_j S_{g(j)}]\right) \\
&= \left(\sum_{i=1}^m d_i\Big(\frac{1}{i} + \frac{1}{n-i}\Big)\frac{1}{1+\delta_{i,n-i}}\right)^{-2} \left(\sum_{i=1}^m d_i^2(\phi_i\theta + \rho_{ii}\theta^2) + \sum_{i\neq j} d_i d_j \rho_{ij}\theta^2\right) \\
&= \left(\sum_{i=1}^m d_i\Big(\frac{1}{i} + \frac{1}{n-i}\Big)\frac{1}{1+\delta_{i,n-i}}\right)^{-2} \left(\sum_{i=1}^m d_i^2(\phi_i\theta + \rho_{ii}\theta^2) + 2\sum_{1\leq i\leq j\leq m} d_i d_j \rho_{ij}\theta^2\right)
\end{aligned} \tag{13}$$

Next, the $Var[\hat{\theta}_W]$ is taken from Achaz (2009):

$$\begin{aligned}
Var[\hat{\theta}_W] = \Big(\sum_{i=1}^m \frac{n}{i(n-i)(1+\delta_{i,n-i})}\Big)^{-2} \Bigg(&\theta\Big(\sum_{i=1}^m \Big(\frac{n}{i(n-i)(1+\delta_{i,n-i})}\Big)^2 \phi_i^{-1}\Big) \\
&+ \theta^2\Big(\sum_{i=1}^m \Big(\frac{n}{i(n-i)(1+\delta_{i,n-i})}\Big)^2 \phi_i^{-2}\rho_{ii} \\
&+ 2\sum_{i=1}^m \sum_{j=i+1}^m \phi_i^{-1}\phi_j^{-1} \frac{n}{i(n-i)(1+\delta_{i,n-i})}\frac{n}{j(n-j)(1+\delta_{j,n-j})}\rho_{ij}\Big)\Bigg)
\end{aligned} \tag{14}$$

Finally, the covariance of $\hat{\theta}_\beta^*$ and $\hat{\theta}_W$:

$$\begin{aligned}
Cov[\hat{\theta}_\beta^*, \hat{\theta}_W] &= Cov\left[\frac{\sum_{i=1}^m d_i S_{g(i)}}{\sum_{i=1}^m d_i(\frac{1}{i} + \frac{1}{n-i})\frac{1}{1+\delta_{i,n-i}}}, \frac{\sum_{i=1}^m S_{g(i)}}{\sum_{i=1}^m (\frac{1}{i} + \frac{1}{n-i})\frac{1}{1+\delta_{i,n-i}}}\right] \\
&= \frac{1}{\sum_{i=1}^m d_i(\frac{1}{i} + \frac{1}{n-i})\frac{1}{1+\delta_{i,n-i}}} \frac{1}{\sum_{i=1}^m (\frac{1}{i} + \frac{1}{n-i})\frac{1}{1+\delta_{i,n-i}}} Cov\Big[\sum_{i=1}^m d_i S_{g(i)}, \sum_{i=1}^m S_{g(i)}\Big] \\
&= \frac{1}{\sum_{i=1}^m d_i(\frac{1}{i} + \frac{1}{n-i})\frac{1}{1+\delta_{i,n-i}}} \frac{1}{\sum_{i=1}^m (\frac{1}{i} + \frac{1}{n-i})\frac{1}{1+\delta_{i,n-i}}} \sum_{i=1}^m \sum_{j=1}^m d_i Cov[S_{g(i)}, S_{g(i)}] \\
&= \frac{1}{\sum_{i=1}^m d_i(\frac{1}{i} + \frac{1}{n-i})\frac{1}{1+\delta_{i,n-i}}} \frac{1}{\sum_{i=1}^m (\frac{1}{i} + \frac{1}{n-i})\frac{1}{1+\delta_{i,n-i}}} \sum_{i=1}^m \sum_{j=1}^m d_i \rho_{ij}\theta^2
\end{aligned} \tag{15}$$

## 5 Standardized $\beta$ statistics

The standardized $\beta$ statistics are given by:

$$\beta^{(1)}_{std} = \frac{\beta^{(1)}}{\sqrt{Var[\beta^{(1)}]}} = \frac{\hat{\theta}_\beta - \hat{\theta}_W}{\sqrt{\alpha^*_n \hat{\theta} + \beta^*_n \hat{\theta}^2}} \tag{16}$$

$$\beta^{(1)*}_{std} = \frac{\beta^{(1)*}}{\sqrt{Var[\beta^{(1)*}]}} = \frac{\hat{\theta}^*_\beta - \hat{\theta}_W}{\sqrt{Var[\hat{\theta}^*_\beta] + Var[\hat{\theta}_W] - 2Cov[\hat{\theta}^*_\beta, \hat{\theta}_W]}} \tag{17}$$

$$\beta^{(2)}_{std} = \frac{\beta^{(2)}}{\sqrt{Var[\beta^{(2)}]}} = \frac{\hat{\theta}_\beta - \hat{\theta}_D}{\sqrt{Var[\hat{\theta}_\beta] + \left(\frac{1}{\frac{T}{2N_e} + \frac{1}{n}}\right)^2 \left(\hat{\theta}^2 + \frac{T\hat{\theta}}{2N_e} + \frac{\hat{\theta}}{n} + \hat{\theta}^2 \sum_{i=2}^{n} \frac{1}{i^2(i-1)^2}\right)}} \tag{18}$$

where $T$ is the estimated speciation time in generations, $N_e$ is the estimated effective population size of the ingroup species, and $\hat{\theta}$ is the estimated mutation rate. For simplicity, we assume that Cov$[\hat{\theta}_\beta,\ \hat{\theta}_D]$=0. This assumption results in a slight underestimate of the variance of $\beta^{(2)}$, as would be expected due to the small negative covariance between the ingroup coalescence time and the number of substitutions. However, under an equilibrium model (constant population size), we show in **Supplementary Fig. 2** our estimate is close to the actual variance of $\beta^{(2)}$.

The $Var[\hat{\theta}_\beta]$ can be obtained from the formula for variance of a general group of estimators presented in Achaz (2009) for which $\hat{\theta}_\beta$ is a member. $\sigma$ is defined in Achaz (2009) and $d_i$ is the measure of frequency similarity from Siewert and Voight (2017).

$$Var[\hat{\theta}_\beta] = \Big(\sum_{i=1}^{n-1} d_i\Big)^{-2} \left(\theta\Big(\sum_{i=1}^{n-1} d_i^2 i\Big) + \theta^2\Big(\sum_{i=1}^{n-1} d_i^2 i^2 \sigma_{ii} + 2\sum_{i=1}^{n-1}\sum_{j=i+1}^{n-1} ijd_i d_j \sigma_{ij}\Big)\right) \tag{19}$$

## 6 Estimation of the background mutation rate and speciation time

Calculating the variance of each $\beta$ statistic requires knowledge of the underlying mutation rate. We recommend estimating this from the data and as such, several estimators of the mutation rate may be appropriate. If sequencing errors are expected to be rare, then Watterson's estimator is a good choice, as it has very low variance. However, in practice, rare variants can be prone to false or missing calls. In situations like this, estimators which ignore rare variation may be a better choice. Achaz (2008) proposed an estimator similar to Watterson's estimator which use the number of segregating sites, singletons excluded, to estimate the mutation rate: $\hat{\theta}_{S_{-\xi_1}} = \frac{S_{-\xi_1}}{a_n - 1}$, where $S_{-\xi_1}$ is the number of segregating sites excluding singletons and $a_n = \sum_{i=1}^{n} \frac{1}{n-1}$. Achaz (2008) introduces a similar estimator which excludes singletons for when only a folded site frequency spectrum is available. We suggest using one of these two estimators if you believe singletons in your data are prone to false positive or missing calls (e.g., elevated error rates from technology or low-pass sequencing coverage).

The mutation rate can either be estimated at a genome-wide level or for individual loci. Estimating at a locus-by-locus level allows the variance to reflect changes in mutability or background selection. However, doing so can also increase the variance of the mutation rate estimator, as the size of window used to estimate the mutation rate will be smaller. If too small of a window is used, the variance of the denominator of the standardized statistics may swamp signals of selection from the numerator, decreasing power. In practice, we recommend using the largest window you think still reflects local changes in mutation rate that will be important. Using simulations of human parameters, we find that Watterson's theta on 1kb windows surrounding the core SNP does a poor job of estimating the background mutation rate (data not shown), while 10kb windows do significantly better **(Supplementary Fig. 4)**.

The variance of $\beta^{(2)}$ is also dependent on the speciation time (in coalescent units, i.e. units of $2N_e$). The speciation time can be obtained from prior demographic analyses of the species of interests, or by estimating it from the data at hand. The software presented in DeGiorgio *et al.* (2014) implements an estimator of divergence based on the site frequency spectrum and the number of substitutions, which we recommend when prior estimates of speciation time are not available.

# 7 Method of power analysis

**Simulations.** We conducted simulations using the software package SLiM (Haller and Messer, 2017), with identical parameters to Siewert and Voight (2017). Briefly, two species were modeled, corresponding to human and chimpanzee. Each species had a size of 10,000 diploid individuals and the two species had a split time 250,000 generations prior to sampling. In each balanced simulation, an overdominant mutation was introduced in the middle of the simulated 10kb window in species one, either at 250,000 generations or 100,000 generations prior to sampling. If the balanced mutation was lost due to drift, the simulation was repeated until maintenance of the mutation was achieved. We simulated three different equilibrium frequencies: 0.25, 0.5 and 0.75, which correspond to dominance coefficients of $-0.5$, 100 and 1.5, and were paired with selective coefficients of $-0.01$, 0.01 and 0.01. A mutation rate and recombination rate of $2.5 \times 10^{-8}$ was used except when noted. 100 haploid individuals were sampled from species one, and one individual was sampled from species two.

**Power comparison.** Two techniques for power analysis have been used in the literature. We use both, and show that the relative performance of the various methods remains roughly consistent across comparison methods.

I *Single target frequency.* This power comparison method answers the question "How well do the methods distinguish between a balanced SNP at a certain frequency and a neutral SNP of a similar frequency?"

To perform this power analysis, we directly scored the simulated balanced SNP for each statistic. We note that the balanced SNPs are usually not at exactly the equilibrium frequency, due to genetic drift and sub-sampling of individuals, but most are within 10% of the equilibrium frequency (data not shown). For this reason, for the neutral scores, we found a SNP in each neutral simulation replicate within frequency 10% of the equilibrium frequency of the balanced SNPs, and used its score. If there was not a SNP within frequency 10%, we did not use that simulation replicate in that power analysis. In this way, all methods are testing for allelic class build-up at approximately one frequency. Instead of using the frequency of the core SNP, the $NCD2$ statistic requires the user to specify a target frequency (Cheng and DeGiorgio, 2018)(Bitarello *et al.*, 2018). We used a value equal to the expected equilibrium frequency in the balanced simulations, which represents the best case for $NCD$. This power comparison method was used for all figures except **(Supplementary Fig. 6)**.

II *Multiple target frequencies.* This power comparison method answers the question "How well do the methods distinguish between a window with a balanced SNP and any window without a balanced SNP?"

The maximum value of each statistic is used in each simulated balanced and neutral window. However, the values of $NCD2$ calculated using different target frequencies are not comparable. To address this issue, Cheng and DeGiorgio (2018) developed $NCD_{mid}$, which calculates a modified NCD2 value using a grid of target frequencies. We compare the power of NCD using $NCD_{mid}$ for this comparison type. This power comparison method was used for **(Supplementary Fig. 6)**.

For both types of power analysis, we used a window size of 1kb for all summary statistics, as we found this was optimal for all methods **(Supplementary Fig. 6)** (Siewert and Voight, 2017). Only SNPs greater than 2kb from the edge of the 10kb simulation window were used so that a full 1kb window or 20 informative sites

around each SNP was present. For $T1$ and $T2$, we used 10 informative sites on either side of the balanced SNP, which is expected to be a region of about 1kb (Siewert and Voight, 2017; Cheng and DeGiorgio, 2018). To estimate the divergence time for use in the $T1$ and $T2$ statistics, we concatenated the neutral simulations and used the function provided in the BALLET software, which implements $T1$ and $T2$ (DeGiorgio *et al.*, 2014). We gave BALLET the recombination and mutation rate used in the simulations. In the power analysis in which mutation rate varies, the divergence time was estimated using only simulation replicates with the $2.5 \times 10^{-8}$ mutation rate.

# 8 Discussion of power analysis results

**Comparison with prior power analyses.** In both **Supplementary Fig. 5** and **Supplementary Fig. 6**, our results coincide with those of Cheng and DeGiorgio (2018) in that $T2$ outperforms $NCD2$ or $NCD_{mid}$. However Cheng and DeGiorgio (2018) did not compare the power of $\beta$, as they were focused on methods to detect selection shared between multiple species, for which $\beta$ is not especially tailored.

The performance of the $\beta$, $T1$, and $T2$ statistics relative to $NCD2$ deviate from that found in Bitarello *et al.* (2018). This is due to two differences between our power analysis methods. The first, as pointed out in Cheng and DeGiorgio (2018), is that 100, not 10, informative sites were used to calculate $T1$ and $T2$ in Bitarello *et al.* (2018), reducing the power of $T1$ and $T2$. Like in Cheng and DeGiorgio (2018), we used 10 informative sites so the window sizes for all statistics are as equivalent as possible.

Secondly, in Bitarello *et al.* (2018), the value of NCD2 used to calculate power is based on a single core/target SNP frequency. In contrast, the $T$ and $\beta$ values that were used were the maximum $T$ or $\beta$ score across all SNPs in the window. Because $T2$ and $\beta$ adapts to use the frequency of each SNP it is calculated on, this is equivalent to using power comparison method (I) for NCD2, but power comparison method (II) for the other statistics. This increases the number of core SNP frequencies that the $\beta$ and $T$ statistics must test, and therefore artificially increase the false positive rate relative to NCD2. When we use the same power comparison method for all statistics, whether it be using a single target/core frequency or all allele frequencies in the simulated window, we find that $T2$ and the $\beta$ statistics tend to perform the strongest **(Supplementary Fig. 5, Supplementary Fig. 6)**. $T2$ uses simulated site frequency spectra under balancing selection and neutrality. When the computational power, outgroup sequence and knowledge of demographic parameters exist to perform these simulations, our findings suggest that $T2$ may be the ideal statistic to use, while the $\beta$ statistics may be best to use otherwise.

**Comparison of $\beta^{(2)}$ and $NCD2$ statistics.** The $NCD2$ statistic measures the average frequency difference between SNPs in a window and a target frequency, with substitutions considered as SNPs of frequency 0.

$$NCD(tf) = \sqrt{\frac{\sum_{i=1}^{n}(p_i - tf)^2}{n}} \tag{20}$$

where $p_i$ is the allele frequency of the $i$th of $n$ SNPs in a window. The target frequency is analogous to the core SNP frequency from $\beta$ or $T1/T2$. However, NCD requires this parameter to be set by the user, unlike with $\beta$ or $T1/T2$, which use the frequency of the SNP at the center of each window as the target/core SNP frequency. The reason for this is that the expected value of NCD is not constant across target frequencies, so NCD scores can only be compared to scores using the same target frequency.

$NCD2$ and $\beta^{(2)}$ are similar in their approach, in that they both explicitly capture excessive allele frequency correlation. We posit that the relative strength of $\beta^{(2)}$ compared to $NCD2$ is due to several factors. The first is that by using a difference of two unbiased estimators of the mutation rate, $\beta^{(2)}$ has a constant expected value (zero), whereas the expected value of $NCD2$ varies with target frequency (Bitarello *et al.*, 2018). This enables $\beta$ values to be compared across different allele frequencies, so that it can use the exact frequency of

the core SNP, instead of having to use the same target frequency across all SNPs.

Secondly, instead of taking the square of the *average* frequency difference between each SNP and the target frequency, $\beta$ is a function of the *sum* of the frequency similarity. This means that SNPs at large frequency differences away from the core site frequency have very little effect on $\beta$. In contrast, for $NCD$, these SNPs factor into the average and add noise. For instance, NCD with a target frequency of 50 will return the same value if there is a window with ten SNPs at frequency 50% and ten singletons as it will in a window with two SNPs at frequency 50% and two singletons. In contrast, the $\beta$ score will be nearly five times higher in the first case than the second as there is a five times stronger signal of allelic class build-up. In addition, rare variant calls are often problematic in real data and are not indicative of the presence or absence of balancing selection, so an ideal statistic would not be influenced by their presence.

Thirdly, because it does not take into account speciation time like $T2$ or $\beta^{(2)}$ does, the distribution of NCD2 is heavily dependent on speciation time. Too long of speciation time would increase the number of substitutions considerably and could dwarf a signal in the polymorphism portion of the spectrum.

Lastly, NCD2 considers substitutions to be SNPs of frequency zero, which can cause a false signal of excessive allele frequency correlation when trying to detect balancing selection at extreme equilibrium frequencies (Cheng and DeGiorgio, 2018). We instead consider them in a separate estimator, $\hat{\theta}_D$.

# 9 Application to human

We first generated input files for BetaScan using the glactools toolkit with the 1000 Genomes YRI, CEU and CHB population vcf files. The –misroot and –useroot flags were used in glactools to polarize alleles using the inferred allele at the root node.

BetaScan was then run with a window size of 2000 basepairs, a divergence time, $T$, of 12.5 and the default value of $p$ of 2. To produce a map of mutation rate, we calculated an estimator of $\theta$ which excludes singletons from Achaz (2008). We chose to use a 10kb window instead of 2kb to estimate the background mutation rate in order to reduce noise. Using bedtools, we filtered the results for core SNPs which were present in the 1000 genome strict quality filter mask and at least half of the 2kb window was present in the 1000 genome pilot mask.

In order to test if $\beta$ scores are predictive of a SNP being in the GWAS catalog, we first generated a list of GWAS SNPs. Since the lead SNP in the GWAS catalog may not be the true causal SNP, we used plink to find all SNPs within an $r^2$ of 0.9 and 50kb of all SNPs in the GWAS catalog. We then overlapped this list of GWAS SNPs and their proxies with our $\beta$ Scores. In our analysis of $\beta$ and $T2$ with trans-haplotypes, we define rare variants as those below 5%, and use SNPs which passed our quality masks.

# References


Achaz, G. 2008. Testing for neutrality in samples with sequencing errors. *Genetics*, 179(3): 1409–24.

Achaz, G. 2009. Frequency spectrum neutrality tests: one for all and all for one. *Genetics*, 183(1): 249–58.

Bitarello, B. D., de Filippo, C., Teixeira, J. C., *et al.* 2018. Signatures of Long-Term Balancing Selection in Human Genomes. *Genome Biology and Evolution*, 10(3): 939–955.

Cheng, X. and DeGiorgio, M. 2018. Detection of shared balancing selection in the absence of trans-species polymorphism. *Molecular Biology and Evolution*.

DeGiorgio, M., Lohmueller, K. E., and Nielsen, R. 2014. A model-based approach for identifying signatures of ancient balancing selection in genetic data. *PLoS genetics*, 10(8): e1004561.

Haller, B. C. and Messer, P. W. 2017. SLiM 2: Flexible, Interactive Forward Genetic Simulations. *Molecular biology and evolution*, 34(1): 230–240.

Hey, J. 1991. The structure of genealogies and the distribution of fixed differences between DNA sequence samples from natural populations. *Genetics*, 128(4).

Hudson, R. R., Kreitman, M., and Aguadé, M. 1987. A Test of Neutral Molecular Evolution Based on Nucleotide Data. *Genetics*, 116(1): 153–159.

Siewert, K. M. and Voight, B. F. 2017. Detecting Long-Term Balancing Selection Using Allele Frequency Correlation. *Molecular Biology and Evolution*, 34(11): 2996–3005.

Tajima, F. 1989. Statistical Method for Testing the Neutral Mutation Hypothesis by DNA Polymorphism. *Genetics*, 123(3): 585.

Tavaré, S. 1984. Line-of-descent and genealogical processes, and their applications in population genetics models. *Theoretical population biology*, 26(2): 119–64.

Watterson, G. 1975. On the number of segregating sites in genetical models without recombination. *Theoretical Population Biology*, 7(2): 256–276.
